# Targeted Drug Delivery by Gemtuzumab Ozogamicin: Mechanism-Based Mathematical Model for Treatment Strategy Improvement and Therapy Individualization

**DOI:** 10.1371/journal.pone.0024265

**Published:** 2011-09-07

**Authors:** Eva Jager, Vincent H. J. van der Velden, Jeroen G. te Marvelde, Roland B. Walter, Zvia Agur, Vladimir Vainstein

**Affiliations:** 1 Institute for Medical BioMathematics, Bene Ataroth, Israel; 2 Department of Immunology, Erasmus MC, University Medical Center Rotterdam, The Netherlands; 3 Clinical Research Division, Fred Hutchinson Cancer Research Center, Seattle, Washington, United States of America; 4 Division of Hematology, Department of Medicine, University of Washington, Seattle, Washington, United States of America; 5 Optimata, Ltd, Ramat Gan, Israel; Vrije Universiteit, The Netherlands

## Abstract

Gemtuzumab ozogamicin (GO) is a chemotherapy-conjugated anti-CD33 monoclonal antibody effective in some patients with acute myeloid leukemia (AML). The optimal treatment schedule and optimal timing of GO administration relative to other agents remains unknown. Conventional pharmacokinetic analysis has been of limited insight for the schedule optimization. We developed a mechanism-based mathematical model and employed it to analyze the time-course of free and GO-bound CD33 molecules on the lekemic blasts in individual AML patients treated with GO. We calculated expected intravascular drug exposure (I-AUC) as a surrogate marker for the response to the drug. A high CD33 production rate and low drug efflux were the most important determinants of high I-AUC, characterizing patients with favorable pharmacokinetic profile and, hence, improved response. I-AUC was insensitive to other studied parameters within biologically relevant ranges, including internalization rate and dissociation constant. Our computations suggested that even moderate blast burden reduction prior to drug administration enables lowering of GO doses without significantly compromising intracellular drug exposure. These findings indicate that GO may optimally be used after cyto-reductive chemotherapy, rather than before, or concomitantly with it, and that GO efficacy can be maintained by dose reduction to 6 mg/m^2^ and a dosing interval of 7 days. Model predictions are validated by comparison with the results of EORTC-GIMEMA AML19 clinical trial, where two different GO schedules were administered. We suggest that incorporation of our results in clinical practice can serve identification of the subpopulation of elderly patients who can benefit most of the GO treatment and enable return of the currently suspended drug to clinic.

## Introduction

Gemtuzumab ozogamicin (GO) is an immunoconjugate between a humanized IgG4 CD33 monoclonal antibody (mAb) and a calicheamicin–γ1 derivative [1]. The target antigen is expressed on myeloid cells as well as on leukemic blasts from more than 80% of AML patients, but is absent on pluripotent hematopoietic stem cells and non-hematopoietic cells [1]. Binding of GO to the CD33 antigen leads to internalization of the drug-antigen complex and hydrolytic release of the toxic calicheamicin component [2]. GO was approved for the treatment of elderly patients with relapsed AML not considered candidates for standard chemotherapy, after demonstration of an approximately 25% overall response rate in this patient population [3]. More recent studies have suggested a benefit of combining GO with other chemotherapeutics [4], and ongoing clinical trials are expected to further define the exact role of GO in AML therapy [5]. However, the optimal schedule and dosing of GO remains unclear [5]. Recent press-release of the drug manufacturing company (Pfizer) determined that the drug is currently withdrawn from the market due to lack of survival benefit and excessive toxicity in SWOG S0106 randomized clinical trial where GO was added to the regular induction treatment in younger AML patient as first line. However, significant efficacy in elderly patients receiving GO as monotherapy or with low dose cytotoxics is still debated. Given the significant toxicities associated with current clinical use of GO, prospective identification of the patients most likely to benefit from GO and determination of the most efficacious and least toxic GO administration schedule is of considerable interest.

Classical population pharmacokinetic (PK) analysis of GO was performed for the standard dose [6], it showed decrease in volume of distribution and clearance rate during second drug infusion, probably due to lowering of the blast burden, which is responsible for specific CD33 mediated drug clearance. However, this standard approach failed to provide the information needed for individualization of the GO dose and administration schedule, as well as for optimal combination with other cytotoxic drugs. Moreover, standard pharmacodynamic analysis of GO is practically impossible due to requirement for repeated bone marrow biopsies, which are unethical in average elderly, fragile and ill GO recipients. Therefore, alternative modelling approaches should be looked for, allowing for more comprehensive analysis with relatively few available experimental data. Rational design of treatment schedules of mAb-based drugs can be accomplished by mechanism-based models [1], [7]. Mathematical models of receptor-mediated internalization have been developed for peptide ligands and their receptors [8], [9], [10], [11], and used to analyze target-mediated drug disposition of non mAb-based drugs [12]. So far, mechanism-based models have been successfully developed for unconjugated mAbs [13], [14], but not for chemotherapy-conjugated mAb-based drugs such as GO. Since conjugated mAb-based drugs are active only upon internalization, the analysis of intracellular drug content dynamics is important for the overall evaluation of drug action.

In this work, we present the analysis of a general mechanism-based model for a conjugated mAb-based drug using experimental and clinical data of GO interactions with leukemic blasts. The main objectives of the study were, firstly, to evaluate individual parameter values of blast-drug interactions in AML patients and determination of their relative significance for the response to treatment, and, secondly, to propose optimized strategies of GO combined with other cyto-reductive chemotherapeutics for future clinical trials.

## Methods

### 1. Primary AML blast cell samples

We analyzed by a mathematical model the data from relapsed AML patients enrolled in the European phase II Mylotarg protocols 0903B1-202-EU and 0903B1-203-EU, which was previously obtained and published [2], [15], [16].

Briefly, peripheral blood was drawn at 0, 3 and 6 hour points after initiation of a 2-hour intravenous infusion of GO at a dose of 9 mg/m^2^. The samples were transferred to the analyzing laboratory by courier. Peripheral blood cells were then isolated by density-gradient, and used for analysis of GO binding and internalization. In addition, density gradient-isolated mononuclear cells containing leukemic blasts were obtained from pre-treatment bone marrow specimens to determine drug efflux activity [16].

### 2. Human AML cell line

The human AML cell line AML193 was cultured as previously described [17]. AML193 cells were incubated with various concentrations of GO, after which CD33 antigen saturation was evaluated by a flow cytometry assay [2], [15].

### 3. Analysis of GO binding, internalization, and efflux

We carried out parameter estimates of the rate constants of the mathematical model (see below) using two sets of data, namely in vitro experiments with the human AML193 cell line as well as primary AML blast cells [2], [15]. In saturating experiments (performed on both AML193 cells and patient-derived peripheral blood cells), cells were incubated at 37°C with a saturating concentration of GO (5 µg/ml) for different periods of time (0, 1, 3 and 6 hours). In non-saturating experiments (performed only on AML193 cells), cells were incubated with different concentrations of GO for 15 minutes at 37°C. After incubation with GO, the amount of membrane-bound drug and maximal drug binding (the latter representing the total amount of cell surface CD33 antigen) were assessed by flow cytometry after incubation with secondary biotinylated anti-human IgG followed by addition of avidin-FITC [2]. Results were expressed as channel values of fluorescence intensity. In order to transform these values into absolute numbers of fluorescent molecules per cell, the flow cytometer was calibrated with commercially available FITC-bearing microbeads (SPHERO^TM^ Rainbow Calibration Particles, Spherotech Inc, Lake Forrest, USA). Numbers of fluorescent molecules per cell were further translated into numbers of CD33 molecules per cell using an appropriate stoichiometric calculation. P-glycoprotein function was cytofluorometrically assessed by efflux of the fluorescent P-glycoprotein substrate, DiOC_2_, and expressed as mean DiOC_2_ fluorescence intensity after dye loading divided by DiOC_2_ intensity after dye efflux [16].

### 4. Mechanism-based model of GO-blast interactions

The following mathematical model of mAb-based drug–leukemic cells interaction was applied to the analysis of the collected data. We denote by A the concentration of free GO, by R - the amount of free cell surface CD33 molecules per cell, and by B - that of drug-bound CD33 molecules per cell. Antibody binding to CD33 antigen is assumed to be reversible, with association and dissociation rates k_b_ and k_u_, respectively. Free CD33 molecules are assumed to be generated at a constant rate R_p_ and to have an internalization rate designated by k_e_. The rate coefficient of antibody–antigen complex internalization is denoted by k_i_. The model is implemented by the following system of ordinary differential equations:
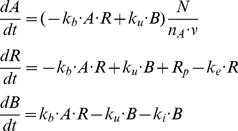
(1)N is the number of blast cells, n_A_ is the Avogadro constant and v is the experimental well volume. Notably, the second and the third equations describe receptor dynamics on the level of an individual cell, while the first equation represents the interaction of the whole leukemic cell population with the drug. The system of equations (1) was used to model in vitro experiments. The appropriate initial conditions are: A(0) = A_0_ (A_0_, initial drug concentration); R(0) = R_p_/k_e_; B(0) = 0. Here R(0) represents the baseline CD33 antigen expression level (i.e., number of cell surface CD33 molecules before GO administration).

**Figure 1:**
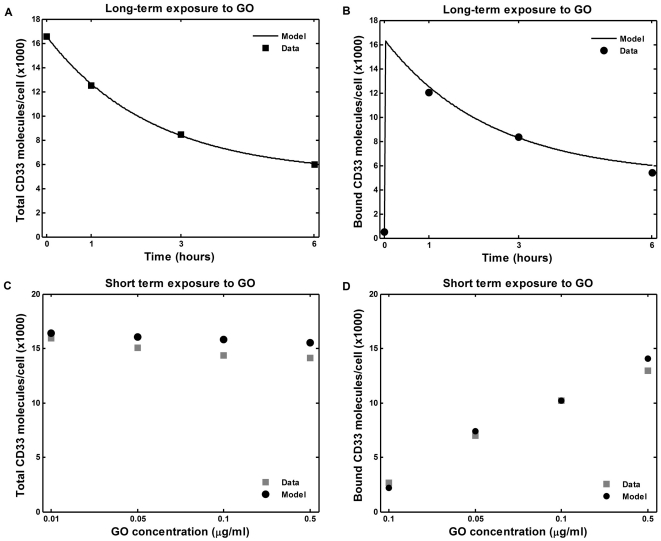
Estimation of the model parameters from in vitro experiments. Model parameters were estimated by fitting model equations to the data of in vitro interaction of GO with AML193 cells. AML193 cells were exposed continuously to either saturating GO concentration (5 µg/ml) for 1, 3, and 6 hours, or to non-saturating GO concentrations (0.1 to 0.5 µg/ml) for 15 minutes. In saturating experiments measured amounts of total (**A**) and bound (**B**) CD33 molecules per cell were fitted by equation (3); CD33 production rate (R_p_), free (k_e_) and bound CD33 internalization rates were estimated. In non-saturating experiments measured amounts of total (**C**) and bound (**D**) CD33 molecules per cell were fitted by equations (1); estimated parameters were drug-CD33 association (k_b_) and dissociation (k_u_) rates (R_p_, k_e_ and k_i_ were set to their values obtained from the saturating experiments). Fitting was performed to the results of single representative experiments.

For the experiments with saturating drug dose following modifications were made to reduce the number of degrees of freedom. If we denote by T the total number of cell surface CD33 molecules per cell (T = R+B), then the sum of the last two equations of (1) yields:
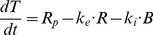
(2)Since in saturation experiments R << B (bound antigens constitute more than 90% of the total, see Figure 1A–B) and k_e_ << k_i_ (i.e., ligand binding facilitates receptor internalization), then B can be approximated by T and k_e_R term is negligible compared to k_i_B term. Thus, we obtain:



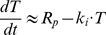
(3)


The initial condition for equation (3) is T(0) = R_p_/k_e._

For the analysis of GO PK in AML patients we used the following system: 
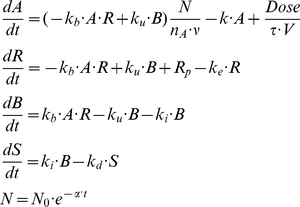
(4)The intracellular content of GO and the rate constant of its elimination by efflux are denoted by S and k_d_, respectively. We assume that the leukemic blast number (designated by N) follows exponential decay, starting 6 hours after the initiation of drug infusion, therefore N = N_0_ for t≤6. These assumptions are based on the results of in vitro experiments where AML193 cells were exposed to GO for different time periods, followed by surviving fraction measurement [2], [15]. N_0_ is the initial blast number and α is the rate of blast elimination by the drug. Non-specific drug elimination rate is designated by k, V is the volume of distribution as evaluated in the absence of leukemic blasts, and τ is the duration of the drug infusion.

### 5. Calculation of drug efflux rate

Efflux of GO from leukemic blast cells was previously estimated using dye efflux [16]. Reported values were ratios of intracellular dye contents (designated by C) at time 0 and 90 minutes post dye loading. Assuming fractional dye efflux dynamics, the following calculation of efflux rate (k_d_) can be used:



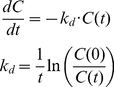
(5)All computer simulations were run on a PC computer with Matlab 7.0 software using ode23 solver. The model was fitted to mean data of GO blood concentration time-courses and to individual data of free and GO-bound CD33 molecules on the peripheral blasts of GO-treated individual patients using nlinfit routine of Matlab 7.0.

## Results

The model consists of the above presented systems of ordinary differential equations (1–5) describing in vitro and clinical experiments. Model parameters include drug-CD33 antigen association and dissociation rates, CD33 antigen production rate, rates of free CD33 antigen internalization and CD33 antigen-drug complex internalization, intracellular drug elimination rate, non-specific drug elimination rate in blood, blast death rate, initial blast burden and volume of drug distribution.

### 1. Evaluation of the model parameters for GO interaction with leukemic blasts

#### 1a. In vitro AML cell line experiments

A saturating concentration of GO (5 µg/ml) was added to cultures of AML193 cells for 1, 3 and 6 hours. The flow cytometry analysis of the cell population at these time-points allows quantitative evaluation of two model variables, namely the mean number of free cell surface CD33 molecules and the mean total number of cell surface CD33 molecules. Using these data and equation (3), we estimated rates of CD33 antigen production, free CD33 antigen internalization and CD33 antigen-drug complex internalization (R_p_, k_e_, and k_i_ respectively). The fit of the model simulations to the experimental measurements is shown in Figure 1.

In separate experiments, AML193 cells were exposed to several non-saturating GO concentrations for a short time period (15 minutes). Subsequently, the other two model parameters (drug-CD33 antigen association (k_b_) and dissociation (k_u_) rates) were estimated using the system of equations (1) with the previously established values for R_p_, k_e_, and k_i_. The resulting fit of the model simulations is shown in Figure 1C,D. The values of all the estimated model parameters are shown in Table 1.

**Table 1:** Estimates of the model parameters.

Parameter designation and meaning	*In vitro* data(AML 193 cell line)	Clinical data(blast cells from AML patients)
**R_p_, CD33 production rate (molecules cell^−1^ hour^−1^)**	2047	279 (109.6)*
**k_e_, Free CD33 internalization rate (hour^−1^)**	0.12	0.213 (65.7)
**k_i_, GO-CD33 complex internalization rate (hour^−1^)**	0.4	0.66 (50)
**k_d_, GO efflux rate (hour^−1^)**	NM	0.34 (64.7)
**k_b_, GO-CD33 association rate (M^−1^ hour^−1^)**	1.1×10^12^	NM
**k_u_, GO-CD33 dissociation rate (hour^−1^)**	397.1	NM
**k, Non specific drug elimination rate (hour^−1^)**	NA	0.01919
**N_0_, Initial total number of blast cells**	NA	2.47×10^12^
**α, Blast elimination rate (hour^−1^)**	NA	0.104
**V, Volume of distribution of GO (L)**	NA	6.2

*results of fitting clinical data are reported as mean, and inter-patient% CV (coefficient of variation)

for those parameters for which estimation in individual patients was possible; NM – not measured;

NA - not applicable; GO – Gemtuzumab Ozogamicin.

#### 1b. In vitro experiments with patients AML blasts

We used GO-blast interaction and drug efflux data from phase II trials of GO monotherapy, [2], [15], [16] in which blood samples were drawn 0, 3 and 6 hours after initiation of GO infusion. In order to be eligible for our analysis, individual patient blood samples had to have discernable blast population and both peripheral CD33 antigen load and efflux data needed to be available. Forty seven of 276 patients who participated in the trial met these criteria and were chosen for further analysis.

Since the standard clinical dose of 9 mg/m^2^, given as 2 hour infusion, causes saturation of CD33 antigenic sites during initial 12 hours [6], we used the same procedure applied for the in vitro saturation experiments to estimate the values for the CD33 production rate as well as free and bound CD33 internalization rates (R_p_, k_e_, and k_i_ respectively) in individual patients. Non-saturating experiments were not performed with primary AML blasts. Consequently, the values of GO-CD33 binding and dissociation (k_b_ and k_u_), estimated from in vitro experiments with AML193 cells, were used for this analysis as well. Drug efflux rates were calculated from dye efflux ratios [16] using equation (5). Estimated parameter values are shown in Table 1.

Notably, we did not confine the analysis to measurement of CD33 expression level (i.e. number of CD33 molecules per cell) but estimated the two processes determining it – namely CD33 production and internalization separately.

### 2. Analysis of GO PK in blood and estimation of initial blast burden

**Figure 2:**
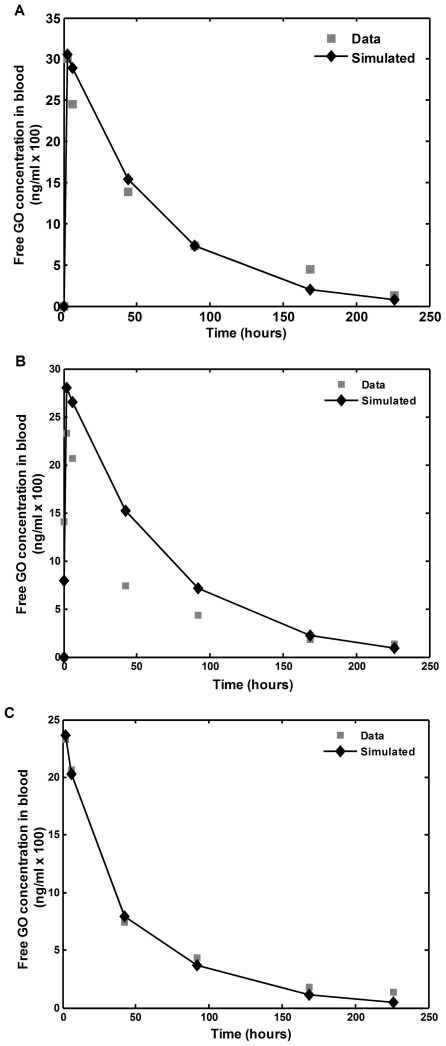
Dynamics of concentration of GO in blood during first and second drug infusions. (**A**) One compartment first order model was fitted to the free drug concentration in blood during second infusion (data taken from [6]). Estimated parameters were non-specific elimination rate (k) and volume of distribution (V). (**B**) The same one compartment model (**A**) is compared to the data of the drug concentration in blood during first infusion. (**C**) The mechanism-based PK model (equations (4)) was used to estimate the initial number of blasts (N_0_) and their death rate (α) using data from the first infusion (k and V were set to their values estimated from the data of the second infusion).

We analyzed the mean blood PK data of GO [6] using our mechanism-based model. Standard administration of the drug included two infusions, separated by a 14-day washout period. We assumed that during the second infusion the number of blasts is negligible and that the drug is cleared only non-specifically, and accordingly used a one-compartment first order kinetics model. This assumption was based on the fact that in 40% of the AML patients, the blast count was less than 5% before the second drug infusion [3], while in most of the remaining 60% of patients, significant decreases in absolute blast population were noted (VHJ van der Velden, unpublished data), and our analysis shows that such a decrease will make the contribution of blast-mediated GO clearance insignificant (see the explanation below). Figure 2A shows the fitting curve of the model to the experimental data of the second drug infusion. The best estimate for the non-specific elimination rate was 0.01919 (1/hour) and that for the volume of distribution was 6.2 (liters). Simulation of the model with these parameter values showed an inadequate fit to the first infusion during an initial phase, but quite a good fit after 4 days (Figure 2B).

After incorporating specific clearance of the drug by leukemic blasts (see system of equations (4)), we fitted the model to the first drug infusion, while varying only the initial number of blasts and their death rate. The values of non-specific clearance and volume of distribution were taken from the analysis of the second drug infusion. All the other parameters were set to population means, as estimated above (Table 1). The resultant fit of the whole model of GO blood PK is shown in Figure 2C. The estimated total body initial number of blasts was 2.47·10^12^ cells.

### 3. Estimation of intracellular exposure to GO and parameter sensitivity analysis

**Figure 3:**
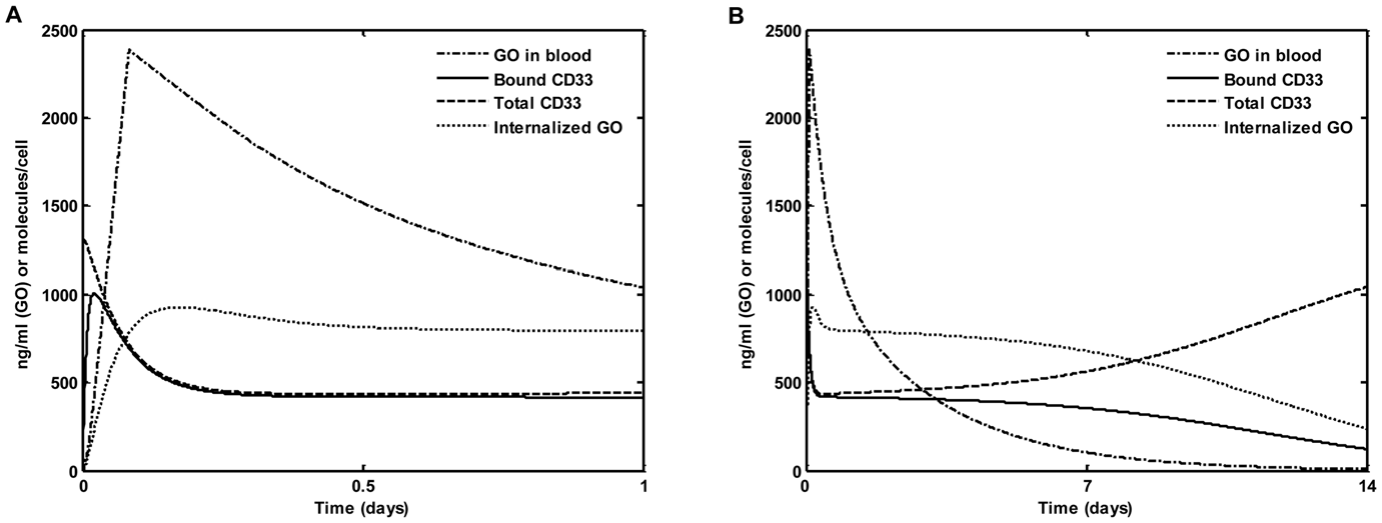
Simulation of the of CD33-drug interaction dynamics in patients. The mechanism-based PK model (4) was used to simulate dynamics of free, cell surface-bound and intracellular drug levels during and after 2-hour intravenous infusion. Model parameter values were set at population means (Table 1). The time series of each variable is depicted for short (**A**) and long (**B**) time period.

**Figure 4:**
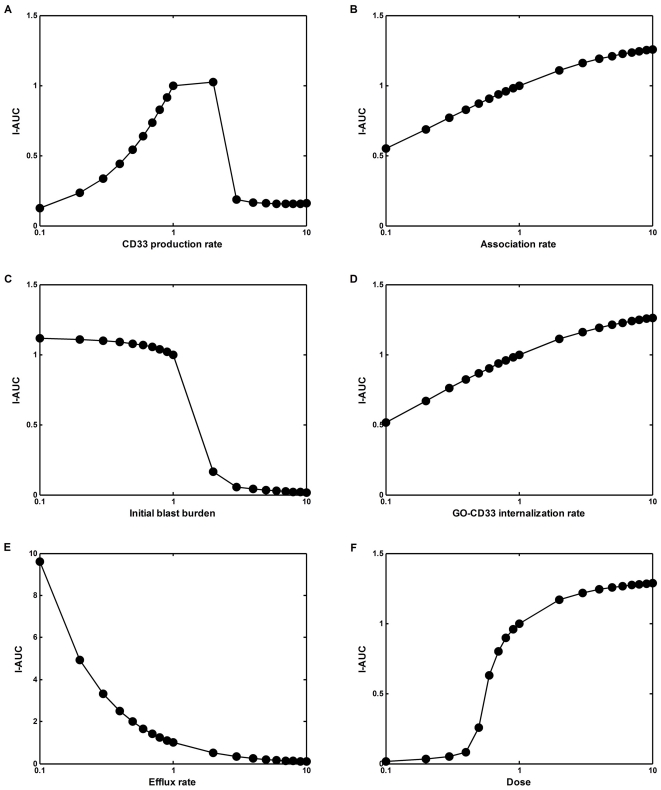
Parameter sensitivity analysis. Intracellular AUC of GO was calculated for different parameter sets. Each time only one parameter was varied, including CD33 production rate (**A**), association rate (**B**), initial blast burden (**C**), internalization rate (**D**), efflux rate (**E**), and total drug dose (**F**). All the other parameters were kept constant at their mean values (Table 1). I-AUC values were normalized to the value obtained for the set of mean parameter values. Parameter values are expressed in relative units after normalization to their mean values.

**Figure 5:**
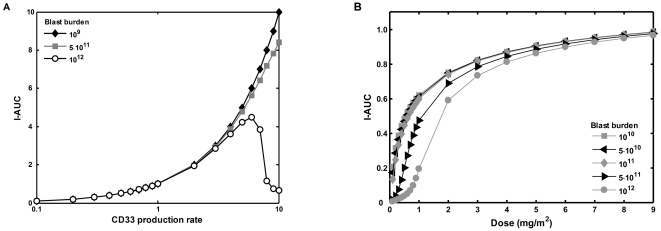
Effect of blast burden on intracellular exposure to GO. Parameter sensitivity analysis for CD33 production rate (**A**) and drug dose (**B**) was performed for various values of the initial blast burden (total number of blasts in the body). I-AUC and CD33 production rate are expressed in relative units as in Figure 4.

Significant differences between extracellular and intracellular GO concentrations could explain a lack of correlation between area under the curve (AUC) of GO in blood and clinical response. While direct measurements of intracellular GO concentrations are currently not feasible since they require repeated bone marrow biopsies, it is possible to calculate the expected intracellular AUC (I-AUC) of the drug based on our model parameter estimations. We used average PK data to perform a sensitivity analysis of I-AUC to different model parameters. To simulate the model behavior, we used the system of equations (4) and parameter values from Table 1. The time series of the simulated blood and intracellular drug concentrations, together with the mean number of total and bound cell surface CD33 molecules using mean parameters values, are shown in Figure 3A,B. The intracellular drug concentration increased sharply after drug application, and then decreased to a transient steady state level followed by a slow decay. To analyze the dependence of I-AUC on different model parameter, parameter values were changed from one tenth to ten times their averages, a range that covers the physiologically relevant values as estimated in individual patients (Table 1). The results of sensitivity analysis are shown in Figure 4. Initial blast burden, drug efflux, and the production rate of CD33 antigen were identified as key parameters influencing I-AUC. A high blast burden and high efflux were both associated with lower I-AUC, as expected. Interestingly, the effect of CD33 antigen production on I-AUC was biphasic: both high and low values of production rate were associated with low I-AUC. The results of the analysis of I-AUC sensitivity to CD33 antigen production for a wide range of initial blast burdens are shown in Figure 5A. Apparently, high CD33 antigen production caused lower I-AUC only if the initial blast burden was higher than 5·10^11^ (cells/liter). For a blast burden smaller than 5·10^11^, high CD33 production was associated with increased I-AUC.

We also analyzed the effect of varying the blast burden on I-AUC for several drug doses. As seen in Figure 5B, I-AUC is quite insensitive to dose variation above 3 mg/m^2^ but sharply decreases below the 3 mg/m^2^ dose.

**Figure 6:**
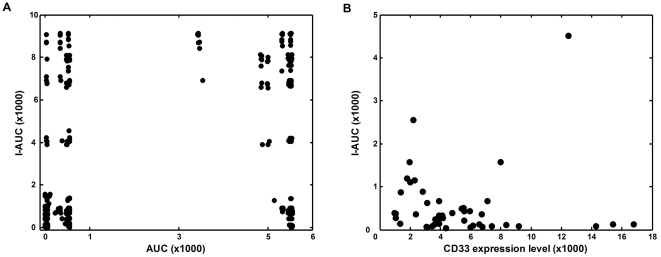
Lack of correlation between calculated intracellular exposure to GO with AUC of the drug in blood and CD33 expression level. The mechanism-based PK model (4) was used to simulate dynamics of free, cell surface-bound and intracellular drug levels during and after 2-hour intravenous infusion. (**A**) Correlation between blood vs intracellular AUC plot obtained by simulating the mechanism-based PK model and using wide range of model parameters (N_0_, Dose, k_b_, R_p_, k_e_, k_i_ set to tenth, one and ten times their average values). (**B**) Baseline CD33 antigen expression level vs. calculated intracellular AUC. Each point represents calculation for an individual AML patient. Variable units: AUC – ng/ml*day, I-AUC – molecules/cell*day, CD33 expression level – molecules/cell.

There was no correlation between the computed values of I-AUC and AUC obtained by varying 6 of the model parameters (N_0_, Dose, k_b_, R_p_, k_e_, or k_i_), for a wide range of parameter combinations (see Figure 6A). Additionally, for the parameter values found in the studied AML patients, there was no correlation between I-AUC and CD33 antigen expression level (i.e. number of CD33 molecules per cell before GO administration) (Figure 6B).

To validate model predictions we analyzed recently published results of EORTC-GIMEMA AML19 clinical trial, where two GO administration schedules were compared, in a prospective randomized fashion: 3 mg/m2 on days 1, 3 and 5 (fractionated arm), or 6 mg/m2 on day 1 and 3 mg/m2 on day 8. The latter schedule was found to be more efficacious [18]. Simulations of these two schedules by our model, using mean population parameters, demonstrated a lower I-AUC for the fractionated arm (12,591 mol*day/cell versus 11,075 mol*day/cell). As GO is active only upon internalization, higher I-AUC may be expected to result in higher efficacy, as indeed appears in the trial. Dynamics shown in Fig. 3 provide a plausible explanation for this result: intracellular GO concentration displays a plateau until day 7, probably due to saturation of the internalization process. Therefore, additional administration of the drug on days 3 and 5 cannot increase the intracellular drug exposure. Efficacy of the 6 mg/m2 dose in this clinical trial also supports our aforementioned results that this dose reduction causes only limited decrease in I-AUC, as compared to the traditional dose of 9 mg/m^2^.

## Discussion

Three main conclusions can be derived from our analysis. First, pharmacokinetics of targeted drug delivery by GO can be accurately modeled using experimental and clinical data on interactions between GO and AML blast cell. Second, high CD33 antigen production rates and low drug efflux are key factors, determining high intracellular GO exposure. Third, even a modest blast burden reduction may increase intracellular GO exposure and allow the clinical use of a reduced GO dose. Taken together, the presented mechanism-based PK model for GO may be useful in prospectively identifying patients that are most likely to benefit from GO-based therapy, thus improving clinical use of GO.

To the best of our knowledge, this is the first research where interaction of a monoclonal antibody-based drug with target cell population was examined in individual patients, assessing PK parameter sensitivity and its significance for individualizing patient treatment schedules. Due to the relatively narrow range of the parameters estimated for different monoclonal antibody-based drugs [12], [13], [14], our conclusions on the relative importance of certain individually-measurable parameters is probably valid for a wide range of drugs. Therefore, we believe that our approach and our results have a broad pharmacological, pharmaceutical and clinical relevance.

We applied a multi-step approach to parameter estimation, which is better suited for systems with parameters of different order of magnitude, as is the case here, than grouping together data taken from experiments of different scales [10]. An AML cell line was employed to measure the previously unreported drug-CD33 antigen association and dissociation rates. Since these rates are determined by chemical processes, they are expected to have limited inter-individual variability, similar to that of primary AML cells. In contrast, CD33 antigen production rate, free and bound CD33 antigen internalization rates, and drug efflux rates, were estimated individually in primary AML specimens, displaying high inter-individual variability, which resulted in a wide range of estimated intracellular exposures to GO.

Intracellular drug exposure (I-AUC) was calculated based on influx of GO through CD33 internalization and efflux through P-glycoprotein. I-AUC is a surrogate for the mean population PD of GO, assuming that high I-AUC is a prerequisite for a significant response to the drug [16]. GO internalization constitutes a first step in the chain of reactions leading to the specific action of calicheamicin on DNA, but GO processing and its interactions with DNA are insufficiently studied on a quantitative basis and cannot be readily assessed in individual patients.

We used previously published data of GO PK in blood for estimating the volume of distribution, non-specific GO elimination rate, initial blast burden and blast elimination rate by GO [6]. The large discrepancy between the estimated volume of distribution and non-specific drug clearance between the first and the second drug infusion, produced by a standard compartmental analysis of GO blood PK, was most likely explained by specific receptor-mediated drug elimination in leukemic blasts [6]. Therefore, we incorporated this elimination mechanism in our model under the assumption of exponentially decreasing numbers of leukemic blasts. Our adjusted model predictions well retrieved the clinical results, implying the significance of specific receptor-mediated drug elimination in the studied process. Moreover, the model-estimated initial blast burden of 10^12^–10^13^ cells in AML patients is similar to that obtained by complex and invasive quantitative histopathologic and radiometric methods [19], [20]. Thus, our model provides a new non-invasive methodology for evaluating leukemia burden, by the analysis of individual blood PK data of conjugated or unconjugated mAb, with a significant receptor-mediated elimination.

The fact that only mean PK data were available to us posed no limitation to our study, since one of our major conclusions is that non-specific drug clearance, peak blood concentration and AUC are not correlated with the estimated intracellular drug exposure (Figure 3C). Note that the AUC range evaluated in our study was 5 times larger than the one found in leukemia patients [6].

Previous analyses of clinical GO monotherapy trials show that many factors determine sensitivity of AML blasts to GO, and account for the considerable variability of clinical responses [21]. Notably, multi-drug resistance (MDR) mediated by P-glycoprotein correlated with clinical response but divergent outcomes in AML patients with low MDR activity, and lack of efficacy of MDR inhibitors, indicate that additional factors may be of importance [16]. In a uni-variate analysis, CD33 antigen expression level (i.e., the number of cell surface CD33 antigen molecules before drug administration) was associated with favorable clinical response, but did not correlate with the response after adjustment for MDR activity [16]. By comparison, GO-CD33 antigen complex internalization rate and blood drug AUC showed no correlation with clinical response [2], [6], [15]. The latter finding could be explained by our model simulations. These show that intracellular drug levels rapidly reach a plateau, which persists for a relatively long period. In contrast, the free GO concentration in blood sharply decreases, concluding that there is no correlation between I-AUC and blood AUC.

Parameter sensitivity analysis showed that three parameters were critical determinants of I-AUC, namely drug efflux, CD33 antigen production rate, and initial tumor burden, all other parameters being much less influential. These results are corroborated by the aforementioned clinical studies [2], [6], [16] showing that internalization rate and AUC in blood do not influence response to GO. It can be stated that the importance of MDR activity and CD33 production rate on the intracellular exposure to GO is clear from the drug's mechanism of action, but it is impossible to predict that *only* these two parameters out of nine possibilities are of major weight without mathematical modeling of the GO-blast interactions.

Our model analysis shows that blast burden significantly influences I-AUC. At low blast burden (less than 5·10^11^), I-AUC was found to be linearly correlated with CD33 antigen production rate. At high blast burden I-AUC was low, both under high and under low CD33 antigen production rates. These effects can be explained by increased blast-mediated specific drug elimination. It is important to note that our simulations failed to show a correlation of the CD33 antigen expression levels, *per se*, with either the estimated CD33 antigen production rates, or with I-AUC. This is explained by the fact that CD33 antigen expression levels depend on both CD33 antigen production rate and on free CD33 antigen internalization rate. These observations are supported by recent studies in engineered AML cell lines [22]. Significantly, our simulations demonstrate that by lowering the initial blast burden the I-AUC is increased. It is, thus, tempting to speculate that reduction of the blast burden by other chemotherapeutics could improve GO efficacy. Unfortunately, individual PK data were not available for the patients analyzed in our study, and, therefore, individual I-AUCs could not be computed. Additional clinical trials are required for validating the proposed use of CD33 antigen production rate, and initial tumor burden as biomarkers of the response to GO.

The lowest effective GO dose, either as a single agent, or in combination with other chemotherapeutics, is still unknown. Our model indicated that increasing the GO dose beyond the standard 9 mg/m^2^ [23] does not increase the I-AUC any further, while decreasing the dose lowers the I-AUC. Nevertheless, for a wide range of initial blast burdens, the difference in I-AUC between a dose of 4 or 9 mg/m^2^ is less than 20%. Moreover, for a lower blast burden (less than 5·10^11^), GO dose can be further reduced to 3 mg/m^2^ with only a 15% decrease in I-AUC.

Our simulation results indicate that the underlying model appropriately describes GO PK and its interaction with CD33: 1) the model fits well both blood PK data and the number of free and bound CD33 molecules on blasts following drug administration; 2) the estimated mean initial leukemic blast burden is close to published values [19], [20]; and 3) the parameter sensitivity analysis is congruent with previously published clinical studies.

One limitation of our research was reliance on peripheral blood blast analysis, rather than on bone marrow data. Since general conclusions of our analysis are valid for a wide range of parameters, they probably would not be altered after incorporation of bone marrow data.

In conclusion, our model predicts that patients who have low MDR activity and a high CD33 production rates are most likely to benefit from GO. These two parameters can be evaluated relatively easily by flow cytometry, after in vitro exposure of blast cells to GO. Our findings further suggest that GO efficacy could be enhanced when used after the leukemic tumor burden was modestly lowered, e.g by alternative cyto-reductive agents. Model predictions suggest that the second GO dose should not be administered before day 7, which is corroborated by comparison with data from prospective randomized trial of GO in elderly AML patients [18].

We suggest that incorporation of our results in clinical practice can serve identification of the subpopulation of elderly patients who can benefit most of the GO treatment and enable return of the currently suspended drug to clinical armamentarium of hematologists.
